# Laser restructuring and photoluminescence of glass-clad GaSb/Si-core optical fibres

**DOI:** 10.1038/s41467-019-09835-1

**Published:** 2019-04-17

**Authors:** S. Song, K. Lønsethagen, F. Laurell, T. W. Hawkins, J. Ballato, M. Fokine, U. J. Gibson

**Affiliations:** 1 Department of Physics; PoreLab, Norwegian University of Science and Technology, N-7491 Trondheim, Norway; 2 Department of Applied Physics, KTH Royal Institute of Technology, Stockholm, 10044 Sweden; 3 Department of Materials Science and Engineering, Clemson University, Clemson, SC 29634 USA

**Keywords:** Materials for optics, Semiconductor lasers, Optical materials and structures, Characterization and analytical techniques

## Abstract

Semiconductor-core optical fibres have potential applications in photonics and optoelectronics due to large nonlinear optical coefficients and an extended transparency window. Laser processing can impose large temperature gradients, an ability that has been used to improve the uniformity of unary fibre cores, and to inscribe compositional variations in alloy systems. Interest in an integrated light-emitting element suggests a move from Group IV to III-V materials, or a core that contains both. This paper describes the fabrication of GaSb/Si core fibres, and a subsequent CO_2_ laser treatment that aggregates large regions of GaSb without suppressing room temperature photoluminescence. The ability to isolate a large III-V crystalline region within the Si core is an important step towards embedding semiconductor light sources within infrared light-transmitting silicon optical fibre.

## Abstract (web summary)

Semiconductor-core optical fibres are of interest for their non-linear optical and electro-optical properties. Here, GaSb/Si composite-core optical fibres were fabricated and a CO_2_ laser was used to facilitate controlled GaSb segregation within the silicon. This has implications for embedding light sources in IR-transmitting fibers

## Introduction

Semiconductor-core optical fibres are of interest for their nonlinear optical and electro-optical properties, and a variety of demonstration devices have been made, including fibre-based photovoltaic cells, infrared (IR) detectors, and supercontinuum sources^1–5^. In addition to their role in advanced photonic devices, they can serve as a useful platform for fundamental materials science investigations. Large spatial and temporal temperature gradients can be imposed with a laser to anneal, recrystallise or facilitate reactions in the fibre core. Whether fabrication is by high pressure vapour deposition in fibre pores, or the molten-core fibre drawing method, laser post-processing has proven to be a powerful technique for modifying the properties of the core^6–9^. More specifically, significant progress has been reported on the processing of silicon-core fibres, in which optical transmission losses have been reduced^7,9,10^ and the band-gap energy has been altered^6^.

In addition to unary crystalline semiconductor cores, there is increasing interest in binary and ternary^11–14^ systems, where spatial variations in composition and associated band-gap and refractive index may be induced by laser treatment^15–17^. Semiconductor cores with direct band gaps may yield a variety of new optoelectronic devices, such as efficient fibre-based photo-detectors and sources^18,19^. Photoluminescence (PL) has been demonstrated in both II–VI and III–V compound semiconductor core fibres; with the latter system emitting at room temperature^11,14^.

To date, post-fabrication modification of the local core composition, which is of particular interest for creating in-fibre diode junctions and Bragg gratings, has been demonstrated only in mixtures that form solid solutions; e.g., Si–Ge^15,16^. Composite materials in which eutectic (or pseudo-eutectic) phase separation is expected open new possibilities, including abrupt heterojunction formation. One combination of particular interest is a Group IV material and a Group III–V component with a direct, smaller band-gap, which may permit construction of a photon source inside a transmitting host fibre.

Integration of III–V materials with silicon in thin film form (in addition to the more prevalent layered molecular beam epitaxy deposited structures) has been realized using ion implantation of silicon with Group III and Group V ions, followed by annealing^20–22^. The formation of III–V semiconductor nanocrystals in Si was reported, along with Raman and PL properties of the resultant composite films^21–24^. These studies demonstrate the feasibility of using thermally driven phase segregation of a compound semiconductor within a Group IV host, as anticipated by the existence of a pseudo-eutectic phase diagram^25^. While no published phase diagram was found for the ternary system GaSb/Si, both Ga^26^ and Sb^27^ form eutectics with Si with low room-temperature solid solubilities, and Si-GaAs^25^, Ge-GaSb^28^, and Ge-GaAs^29^ form pseudo-eutectics. This suggests that GaSb and Si will likely form a pseudo-eutectic with low mutual solubility. However, larger inclusions than those that can be made by implantation are needed for the fabrication of electrically contacted devices.

In this work, GaSb/Si composite-core optical fibres are fabricated using the molten core drawing method^30^ and a CO_2_ laser is employed to facilitate controlled GaSb segregation within the silicon; a first to the best of our knowledge. As-drawn, the 150 µm diameter semiconductor core is highly oriented and had small lamellar inclusions of the III–V binary GaSb that crystallized in alignment with the surrounding Si. Post-processing with a CO_2_ laser allows spatial segregation of regions of GaSb and epitaxial regrowth of the silicon. These studies illuminate the fundamental materials science of eutectic systems, and suggest a path toward the incorporation of light-emitting regions into semiconductor-core fibres.

## Results

### As-drawn GaSb/Si fibres

**Fig. 1:**
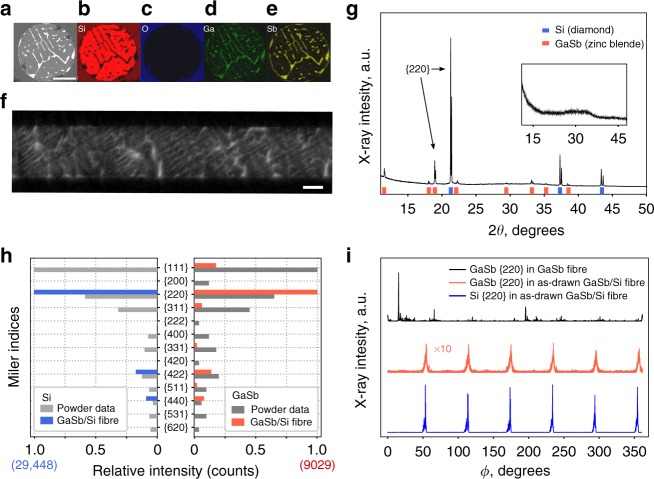
Structural information for an as-drawn composite GaSb/Si fibre. Cross-sectional SEM images of the core; **a** BSE image, and **b** Si, **c** O, **d** Ga and **e** Sb EDX maps showing pseudo-eutectic microstructure (scale bar, 50 µm). **f** X-ray computed tomography image of the fibre (scale bar, 50 µm). **g** X-ray diffraction acquired with fibre rotating around its axis; inset is from a fibre with no core. **h** Relative Bragg intensities observed for silicon and GaSb planes in the composite fibres compared to powder spectra (count number for highest fibre XRD peak in parentheses), and **i** comparison of variation in intensities of the {220} Bragg peaks, during rotation, for a pure GaSb fibre (black), and the GaSb (orange) and silicon (blue) {220} reflections in the composite fibre. Pure GaSb fibres show many reflections, indicating polycrystallinity

Scanning electron microscopy (SEM) confirms the existence of a classic eutectic microstructure with thin interspersed regions of GaSb in a Si matrix. A backscattered electron (BSE) image (Fig. 1a) and energy dispersive X-ray spectroscopy (EDX) maps (Fig. 1b–e) confirm the strong phase segregation of the GaSb and Si, and less than 1 at% oxygen incorporation. Oxygen in the core typically originates from interactions between the molten-core and cladding (oxide) glass during the draw, and low concentrations are critical to achieving phase purity and low optical losses. These reactions can be mitigated for Si, using an interfacial coating^31^. The CaO coating used here does not interact measurably with the GaSb (Supplementary Note 1).

As shown in Fig. 1f, the GaSb inclusions formed during the fibre draw show a high degree of preferential spatial orientation and numerous parallel features, suggesting an ordered crystalline structure. This X-ray computed tomography (XCT) image demonstrates that the eutectic microstructures maintain the same orientation over significant lengths.

X-ray diffraction confirmed the crystalline order in the as-drawn fibres. X-ray scattering intensity as a function of 2*θ* angle from a fibre (during axial rotation) is shown in Fig. 1g, with an inset providing the background spectrum associated with the (silica) glass cladding. Distinct peaks are observed for both cubic Si (*a* = 5.43 Å) and GaSb (*a* = 6.096 Å), as indicated by the blue and orange markers respectively; the double peaks are due to the presence of both the Mo K_α1,2_ X-ray wavelengths from the source. The full-width half maximum (FWHM) of the {220} GaSb K_α1_ peaks (2*θ*) in the as-drawn GaSb/Si composite fibres was 0.055° ± .01, compared to a FWHM of 0.04° for a pure GaSb as-drawn fibre (Supplementary Note 2). Figure 1h shows the crystalline texture of the Si and GaSb in the fibres compared to powder XRD data^32,33^.

The as-drawn fibre cores had reflections from only the Si {220}, {422} and {440} planes, at fibre rotational angle separations indicating that the <111> direction was oriented along the fibre axis. The angular positions of these peaks did not change as a function of position along the length of the fibre tested (Supplementary Note 3) and electron backscattered diffraction (EBSD) confirmed the axial alignment of the silicon (Supplementary Note 4). A near-identical intensity distribution was observed for the GaSb, suggesting that the lattice of the eutectic lamellae in the core interior was epitaxial with that of the Si. Weak {111} and {311} peaks in the GaSb spectrum are associated with misaligned grains.

Figure 1i presents the intensity variations of the {220} Bragg reflections during slow axial (*ϕ*) rotation of the fibres, for a polycrystalline, as-torch-drawn GaSb-core fibre^14^ and for GaSb and Si in the composite-core fibre. For the polycrystalline GaSb fibre (black curve) reflections are seen at a large number of rotational positions, indicating a polycrystalline structure. For the GaSb/Si fibre there are six {220} peaks, separated by 60° in *ϕ*, for each of the constituents (consistent with <111> symmetry). While the Si and GaSb Bragg angles are different for the {220} family, (the orange and blue curves are acquired for different values of 2*θ*), the peaks appear at identical fibre rotation angles, *ϕ*. This demonstrates conclusively that the Si and GaSb lattices share the same orientation; the GaSb solidified epitaxially in the host silicon. The data indicate a single crystalline orientation for this material across the 1.5 cm diameter beam. The same X-ray signature is observed despite the numerous eutectic inclusions that contribute to the GaSb signal, and extends over fibres up to 190 mm in length (the longest sample available). Additional samples exhibited the same orientation, strongly suggesting the fibre core was a single biphasic crystal with some misaligned inclusions (Supplementary Note 4).

The consistency in the angles between lamellae and the fibre axis in XCT images such as (1f) support this observation. In addition, EBSD patterns (Supplementary Note 4) were unchanged when the electron beam was moved between the interior Si and GaSb regions, confirming the epitaxial alignment.

Detailed analysis of the silicon X-ray peak positions indicated tensile strain of about 0.013%, likely associated primarily with the thermal expansion mismatch between the silicon core and silica glass cladding^34^.

### Structure of laser-treated fibres

**Fig. 2:**
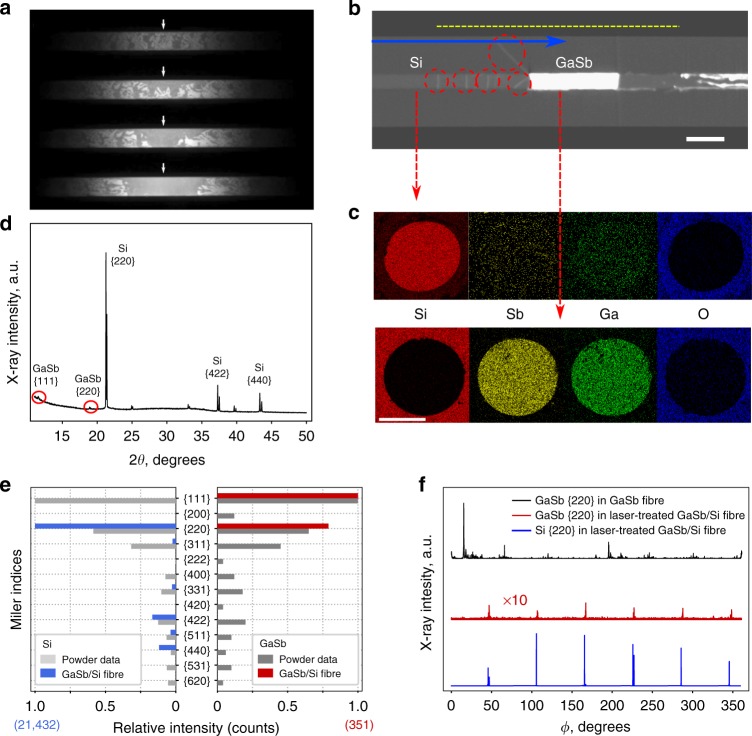
Laser treatment of composite fibres; **a** images of thermal emission during initial heating of the fibre; solid Si is brighter than liquid GaSb/Si alloy due to higher emissivity, **b** XCT of fibre after heating and translation to accumulate GaSb (scale bar 350 µm), **c** EDX elemental maps of silicon and GaSb regions after translation; red arrows show where cross-sections were prepared (scale bar 100 µm), **d** *θ*-2*θ* of the segregated fibre, **e** intensities of X-ray peaks after anneal (blue-Si, red-GaSb), compared to powder files (grey). The maximum numbers of counts for the fibre are in parentheses (**f**) {220} peak intensities during a slow axial rotation

Differences in the emissivity of the two phases made possible studies of laser-induced flow in these fibres. Segregation of the two constituents into large single constituent segments was possible, driven by thermomigration associated with the extreme temperature gradient (~10^4^ K cm^−1^) induced by the CO_2_ laser beam. The melting temperature of GaSb is 712 °C and that for Si is 1414 °C, and the solubility of Si in molten GaSb increases with temperature, so localized heating first melts the GaSb, and the melt then moves by thermomigration through the solid host^15^. This is due to solvation of Si at the high-temperature side of a molten GaSb-rich region, diffusion and subsequent precipitation of silicon at the lower temperature end. A CO_2_ laser was used to aggregate crystals of GaSb in the Si. In the frames in Fig. 2a, where the lighter regions are solid silicon (higher emissivity), the amount of (GaSb-darker) material at the periphery of the heated region was seen to decrease as the liquid flowed to the laser focus. This resulted in a high concentration of GaSb in the molten region compared to the as-drawn fibre. When the laser was scanned relative to the fibre (Supplementary Movie 1), the GaSb-rich liquid remained in the focal region if the melt zone was moved sufficiently slowly. The droplet of GaSb-rich liquid grew as it was drawn through the core and incorporated additional eutectic inclusions. After a single pass, and a slow reduction of power, a GaSb crystal of up to 1.4 mm in length remained at the final laser beam position. The size of the GaSb region was primarily a function of the length of the scanned region and the spot size and power of the CO_2_ laser. The focus determined the temperature gradient, and thus the driving force for thermomigration. If the scan rate was higher than 20 μm s^−1^, GaSb inclusions remained in the silicon because these inclusions could not move through the silicon matrix fast enough to aggregate. Decreasing the laser power too quickly allowed isolation of small inclusions of GaSb in the Si. The 6–10% reduction in laser power was performed in 2% steps, over 30 s, and permitted good segregation of GaSb from the silicon. Quantitative temperature determination from the blackbody emission is challenging in alloy systems, so no accurate cooling curve could be generated.

In Fig. 2b, the blue arrow indicates the laser scan direction, and the yellow dashed bar indicates the size of the melt zone before the laser power reduction, showing the liquid composition was approximately 40 vol% GaSb during the scan. After the laser treatment, GaSb was below the detection limit in the silicon regions and Si impurities in the GaSb were measurable, but less than 1 at%. EBSD results are presented in Supplementary Note 5.

Vertical lines highlighted by the red dashed circles (Fig. 2b) are small regions of GaSb that separated from the larger melt zone during the stepwise laser power reduction, similar to the features seen in vapour-liquid-solid growth during growth-rate fluctuations^35^, and those made during cooling-rate variations in CO_2_ laser annealed SiGe fibres^15^. There is also a crack, filled with GaSb, in the cladding at the edge of the GaSb region, likely due to stress induced during the solidification of the GaSb, which occurred in a single step during (digital) laser power reduction. The density of GaSb decreases from 6.06 to 5.61 g cm^−1^ on solidification (liquid to room temperature), and since the melting point is far below the softening temperature of the cladding, high compressive stresses were developed. For small GaSb inclusions, this could be accommodated, but for any region larger than ~0.3 mm in length, cracking was observed. Cooling rate (within the limits imposed by the power controller) did not affect this result. Cross-sectional EDX maps in Fig. 2c show the uniformity of the silicon and GaSb phase-segregated regions.

Figure 2d is conventional *θ*-2*θ* X-ray diffraction data, and shows the appearance of {111} GaSb reflections. Figure 2e shows the intensities of the silicon and GaSb Bragg peaks relative to powder data^32,33^. The intensities recorded for the GaSb peaks are much reduced over those observed with the eutectic structure; note the smaller number of counts (350) from the most intense peak in the treated fibre, compared to greater than 9000 counts for the as-drawn material (Fig. 1h). The absorption length for the Mo 17 keV X-rays is on the order of 350 µm in silicon—larger than the core diameter, but is only 16 µm for GaSb^36^. With the eutectic microstructure, the X-rays diffracted by the GaSb could escape from deep within the sample, but in the segregated sample, only the surface contributed. The escape depth also affected the apparent texture, as crystal orientations concentrated in the centre of the fibre would not give noticeable signal compared to the silica interface region.

The crystal orientation of the silicon was maintained during processing; X-ray scans on the segregated silicon region showed the same three reflections observed before resolidification; i.e., those from the {220}, {442} and {440} families, as shown in Fig. 2e. Rotational (*ϕ*) scans of the {220} and {422} peaks in the treated region of the fibre showed six occurrences for each family, separated by 60°, indicating retention of the <111> axial orientation. In contrast to the epitaxial behaviour in the as-drawn material, while the segregated GaSb region showed some alignment with the silicon, the appearance of large numbers of <111> peaks demonstrated that the GaSb region was polycrystalline (Supplementary Note 6).

### Photoluminescence

**Fig. 3:**
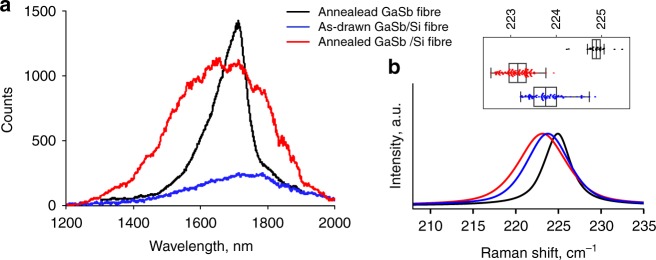
Photoluminescence and strain results. **a** Room temperature emission from an annealed GaSb fibre (black, reduced 2.5 times), with PL from as-drawn (blue) and spot-annealed (red) regions of a composite GaSb/Si fibre. **b** Raman spectra for GaSb the same fibres, showing tensile strain in the composite fibres and peak broadening. The inset shows statistical information on the peak positions; the box width is one standard deviation and the end bars are at three. Coloured dots show the data points

PL was observable at room temperature from the GaSb crystals within the as-drawn fibre, and from regions of the segregated material formed by laser spot annealing, as shown in Fig. 3a. The annealed regions were smaller than the large aggregates used for the materials analysis, so that annealed and unannealed material could be tested from proximal regions of the fibre. The luminescence peak for the eutectic material is substantially broader than that observed for an annealed pure GaSb-core fibre^14^. The red-shift of the PL in as-drawn composite material relative to bulk is consistent with observations of silicon-doped MBE grown GaSb^37^, and the full-width at half maximum linewidth for the GaSb/Si material is about twice that reported for a doping level of 8 × 10^19^ cm^−3^ Si in GaSb films grown on GaAs substrates^38^.

For the spot-annealed fibre, the PL peak was blue shifted by about 50 nm (0.04 eV) from the as-drawn emission, while the linewidth was comparable to that from the eutectic inclusions. The PL signal of the segregated material is of higher intensity, but this may be due to geometrical effects.

Raman results (Fig. 3b) showed a larger range of values centred about a tensile strain-induced shift of ~1.5 cm^−1^ for the composite fibres, compared to the pure GaSb, and additional tensile strain after aggregation of larger GaSb regions. These shifts were comparable to the magnitude of the Raman shift in superlattices of GaSb/AlSb, where strain values of 0.5% were reported^39^.

## Discussion

Fabrication of semiconductor-core glass fibre from the melt, and laser-induced melting and re-solidification of the resultant fibre cores, allows unusual materials processing conditions. The small diameter of the fibres and the laser heating permits establishment of large thermal gradients along the fibre axis, and scanning the fibre promotes rapid solidification. Cooling is promoted by a large (primarily convective) heat loss from small diameter fibres^40^. For single element cores, as-drawn material is typically polycrystalline, and during recrystallization, rapid translation of the heat zone is needed to promote growth of the existing crystalline seed over the growth of competing nuclei^7^.

For alloy-core fibres where the constituents form a solid solution, slower translation of the melt zone is preferred to avoid constitutional supercooling and subsequent segregation of the constituents^15^. Yet slow laser translation still allows single crystal formation because the miscibility gap promotes crystallization of the higher melting point material onto the existing seed, enriching the liquid near the solidifying interface with the lower melting point material. This suppresses the formation of competing nuclei, as the energy barrier for nucleation rises due to the increased separation of the liquid temperature and the solidification point of the precipitating phase. Keeping the temperature gradient large and perpendicular to the growth plane is analogous to the grain selection process used in the casting of superalloy turbine blades^41^.

A pseudo-eutectic system, such as the combination of silicon and GaSb, presents a different dynamic. The recrystallization of silicon onto the existing crystal as the fibre cools is similar to the case of a solid-solution forming alloy, but the silicon that precipitates rejects the GaSb component. Small liquid pockets form, and remain as liquid until the eutectic temperature is reached, below the melting point of pure GaSb (712 °C). The extremely long single crystals observed in the as-drawn material attest to the effectiveness of what is tantamount to a traveling solvent crystallization process for the silicon, but one where the solvent (GaSb) is allowed to crystallize in situ.

The as-drawn GaSb/Si fibres exhibited epitaxial alignment of the constituents, and single crystals over hundreds of mm, with the prevalent orientation being the <111> direction parallel to the fibre axis. The {111} Si planes have the lowest surface energy, and pure silicon-core fibres^42^ are often observed to have this orientation. This is also the preferred growth direction during the vapour-liquid-solid (VLS) process^35,43^, where gold (typically) adopts the role of a traveling solvent as a microwire condenses beneath a small liquid drop.

For the GaSb in the eutectic structure, the liquid was isolated within the silicon-defined “pores” of small dimensions and solidified in situ at the eutectic temperature. Except for some isolated regions, the GaSb crystallized epitaxially on the silicon, with the <111> direction of the lamellae also along the fibre axis.

Using laser-induced melting, the two core phases could be spatially separated into cm-scale Si and mm scale GaSb regions (for the fibre composition studied), with the Group IV crystals retaining the axial <111> orientation. During the laser-induced segregation, Si regrew from the liquid onto the oriented crystalline Si remaining from the fibre drawing process. The GaSb-rich liquid suppressed additional Si nucleation, in a manner similar to that observed previously with GeSi^15^, and single crystal silicon was grown with few competing nuclei. The process resembled the traveling solvent method for crystal growth^44^, with GaSb acting as the solvent. In the laser-driven process, the solvent could be extracted during regrowth of the silicon fibres. This was in contrast to the as-drawn material, where the temperature gradient was insufficient to prevent trapping of GaSb inclusions.

Some degree of polycrystallinity was observed in the GaSb XRD after laser segregation, and EBSD confirmed this. Unlike the silicon recrystallization, where there was a moving solvent to prevent competing nucleation, the GaSb solidification occurred while the laser was held at the final position and the power was reduced, stepwise. At the eutectic temperature, there was quench solidification of the remaining liquid. Although silicon concentrations in GaSb measured using EDX were close to the detection limit, significant strain, which would be expected with even one percent Si in GaSb, was observed in the XRD, PL and Raman spectra. Additional directional recrystallization, performed at a power level that does not melt the silicon, might permit epitaxial regrowth of GaSb on the silicon, with one side of the core serving as a seed.

The demonstration of room temperature PL in these fibres indicated that the growth of the GaSb from the pseudo-eutectic liquid did not introduce sufficient impurities or defects to prevent emission. Peak broadening and the blue shift were attributed to strain. PL spectra of strained III-V systems show an increase in the band gap when there is an excess of smaller ionic radius species^45^ and tensile strain induced by the silicon could result in the GaSb band gap increasing slightly; X-ray FWHM increases demonstrated the introduction of variable levels of strain.

The use of molten-core fibre-drawing to fabricate these novel IV/III–V composite optical fibres highlights the power of this technique for studying novel mixtures of materials. While there is no known published phase diagram for Si-GaSb, the fibre format made the determination that this is a pseudo-eutectic system straightforward. The fibre structure allowed multiple forms of analysis, including the rapid determination of epitaxial growth using X-ray diffraction. Further, because of the (cladding) glass encapsulation, oxidation processes were largely avoided during resolidification and internal structuring.

The as-drawn fibres studied here had a high degree of crystalline order, but a complex microstructure. However, the presence of a second material that formed a (pseudo-) eutectic with the silicon made the equivalent of laser-driven traveling-solvent recrystallization possible while supressing competing nucleation events. This facilitated creation of large single-crystal silicon regions under a wide variety of translation speeds during laser processing, in contrast to the pure silicon case. This result should be quite general, providing a path forward to the production of macroscopic lengths of single crystal cores of semiconductors within a glass cladding for optical, electrical and mechanical applications. Controlled segregation of the components may be used to make light-emitting and other devices within the confines of a single fibre.

## Methods

### Fibre fabrication

Fibre drawing was performed on a custom-designed 6.5 m fibre tower. Preforms were assembled from a 28 mm outer diameter (OD) pure silica glass tube with a central 8 mm bore, into which a second, 7 mm silica tube (inner diameter of 5 mm) was sleeved. The interior surface of the smaller tube was coated with a CaO layer^31^ to minimize reactions between the glass and the molten semiconductor core during molten core fibre formation. This layer does not react chemically with the silicon or GaSb (see Supplementary Note 1). The preforms then were loaded with 94% intrinsic silicon (El-Cat Inc., *ρ* > 20,000 Ω cm) and 6% GaSb (Alfa Aesar, purity 99.99%), expected to be close to the eutectic composition, based on similar systems. Fibres were drawn at a temperature of approximately 1950 °C down to ODs ranging from 0.7 mm to 1.0 mm. The diameter mismatch between inner and outer glass preform tubes led to an air-filled cavity along one side of the drawn fibres in some regions; no difference was seen in the core structure or composition in the presence of this gap.

### Laser post-processing

CO_2_ laser treatment was performed using an experimental configuration similar to that described in ref. ^7^, either with a stationary beam to treat the core locally “spot annealing”, or by scanning the beam relative to the fibre to aggregate GaSb into larger inclusions. Direct observation of the melting and recrystallization were made possible by the step-change in emissivity at the solid-liquid interface^15^. A CCD camera provided real-time feedback on the size of the molten region and the stability of the crystallization front. The largest GaSb regions resulted from translation at a rate of 18.5 µm s^−1^ over a distance of up to 10 mm. After scanning, the laser power was reduced over 10–30 s to minimize solidification-induced stress in the fibre.

### Characterization

Scanning electron microscope (SEM) micrographs were acquired using a Hitachi TM3000 operating at an accelerating voltage of 15 kV in BSE analysis mode. BSE is sensitive to atomic weight, providing ready identification of the GaSb and Si-rich regions in the cores. A Quantax70 Energy dispersive X-ray (EDX) Spectrometer was used for composition determinations. Backscattered electron diffraction (EBSD) was performed using a Hitachi SU6600 with an acceleration voltage of 20 kV, a 70° tilt and the detector at 90° to the excitation beam to confirm the X-ray results on the crystal structure of the fibres.

Raman characterization was made with a Renshaw InVia system with a source wavelength of 785 nm. Photoluminescence (PL) spectra were measured with an excitation wavelength of 1064 nm. Spectra of the as-drawn fibres were taken from both polished cross-sections and through the transparent cladding glass, while the spot-annealed fibre was measured through the glass.

A Bruker DaVinci powder diffractometer with a 15 mm diameter beam from a Mo K_α1,2_ X-ray source (*λ* = 0.7093 Å, 0.7139 Å)^42,46^ was used in this work. The silica is sufficiently transparent at these wavelengths to permit analysis of the crystalline core without removal of the cladding. For crystallographic orientation studies, fibres were mounted in the holder designed for a capillary filled with powder, and the fibre axis was optically aligned perpendicular to the *θ*-2*θ* X-ray scattering plane. After alignment, conventional *θ*-2*θ* scans were taken while rotating the fibre on its axis through 360° at each *θ* step of 0.01°. The major Bragg reflections were identified, and for each of these *θ* values, the signal for all rotational positions (*ϕ*) around the longitudinal fibre axis, was recorded, permitting determination of the orientation of highly crystalline samples (Supplementary Note 7).

XCT was performed using a Bruker Skyscan1172unit, at an acceleration voltage of 167 kV, source current of 59 μA, and image pixel size of 0.65 μm.

## Supplementary information

Supplementary Movie 1

Supplementary Information

Description of Additional Supplementary Files

## Data Availability

The data that support the findings of this study are available from the corresponding author upon reasonable request.
