# Supplementary material for: Laser restructuring and photoluminescence of glass-clad GaSb/Si-core optical fibres
Source: Nat Commun. 2019 Apr 17;10:1790. doi: 10.1038/s41467-019-09835-1 (PMC6470204; doi:10.1038/s41467-019-09835-1)
Supplement: Supplementary file 2 — Supplementary Information [file 41467_2019_9835_MOESM2_ESM.pdf]

Supplementary Information:

Contents

Supplementary note 1*: Calcium Oxide Interface modifier* 2

Supplementary note 2: *XRD of as-drawn GaSb fibre* 3

Supplementary note 3: *Positional X-ray results for as-drawn fibre* 4

Supplementary note 4: *EBSD of as-drawn GaSb/Si core*.. 5

Supplementary note 5: *EBSD of segregated fibre* 6

Supplementary note 6: *{111} peak X-ray results for segregated fibre* 7

Supplementary note 7: *X-ray analysis method using capillary Bragg instrument* ... 8

Supplementary Note 1: Calcium Oxide Interface modifier

The effect of CaO as a protective interface layer between the silicon and the SiO_2 cladding has been established in the literature[2], with reduced oxygen incorporated in the core of fibers, and improved optical transmission.
While GaSb has been reported to react with elemental Ca to form $Ca_5Ga_2Sb_6$[3,4] or $Ca_{11}GaSb_9$[5], these compounds have orthorhombic structures. The XRD spectrum of the fiber core was not compatible with this symmetry, suggesting little if any GaSb-Ca compound formation.
In addition, Ca and O content were below the detection limits (Fig. 1) in Electron Probe Micro-Analysis (EPMA) in the GaSb regions of the as-drawn and annealed fibers. The EPMA map for Ca showed shadows in the region of the core, but a detailed spectrum (Fig. 2) of the Sb signal from this part of the sample, compared to the spectrum of a Ca standard, showed that this is noise, not a real Ca signal
The CaO layer remains well segregated at the interface in the fibers even after the laser treatment, suggesting that GaSb and CaO are not reactive, even at high temperatures.

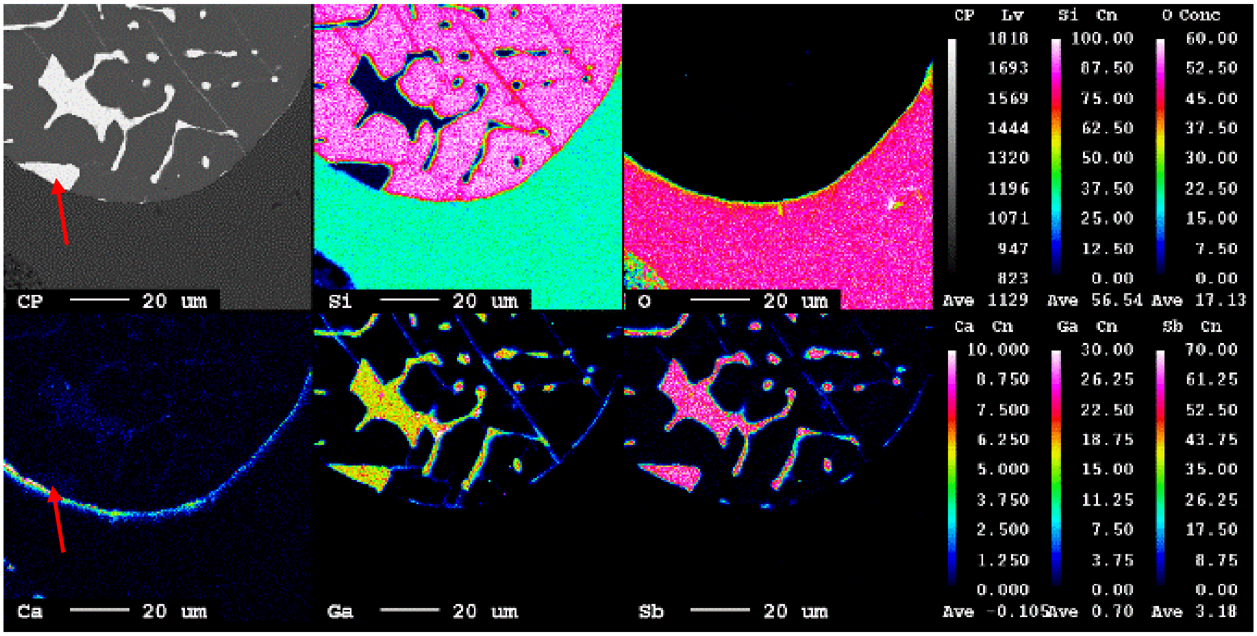

Fig. 1 EPMA results on the as-drawn GaSb/Si core fibers, showing localization of calcium at the interface between the SiO_2 and the semiconductor core. GaSb regions are brighter in the BSE image, and it can be seen that there is no leakage of Ca into the GaSb in the lower left grain indicated by the red arrow.

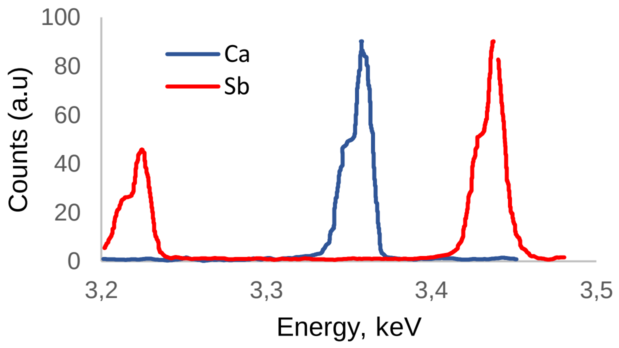

Fig. 2 Comparison of high resolution scan for Sb signal from GaSb region with that of a Ca standard, showing that there is no Ca present in the fiber GaSb, despite the 'shadows' in the EMPA image.

Supplementary Note 2: XRD of as-drawn GaSb fibre

We compared the FWHM of as-drawn pure GaSb-core fibers to the composite, aligned composite fibers to assess the influence of the interfacial strain where the GaSb joins the silicon. The lattice constant of silicon is 0.54 nm, while that of GaSb is 0.61 nm. These results are shown in Fig. 3.

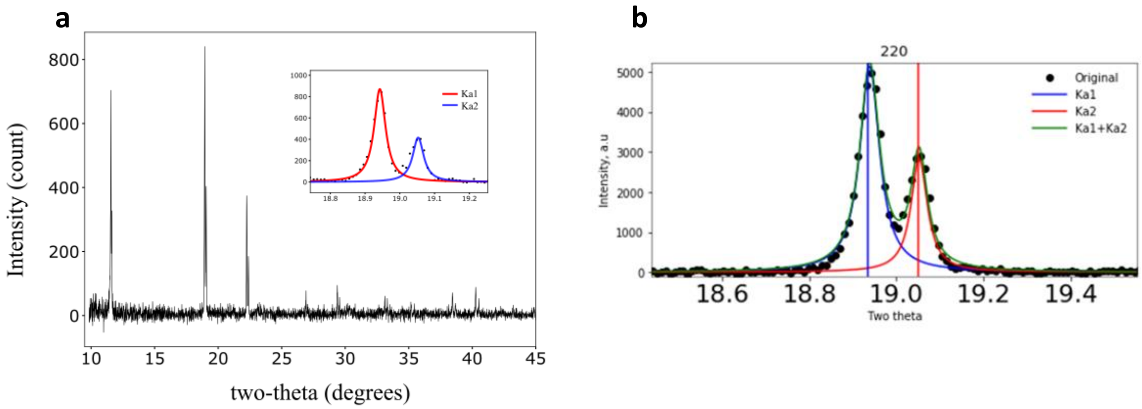

Fig. 3 X-ray diffraction scan for as-drawn fibres. (a) as-drawn GaSb fibre. The inset shows the {220} peaks, and the fit to the $K_{\alpha 1}$ peak yields a FWHM of 0.04 degrees. (b) Detail of {220} scan from as-drawn GaSb/Si fibre. The FWHM of this fibre is 0.055 degrees, demonstrating the strain introduced by the epitaxial growth.

Supplementary note 3: Positional X-ray results for as-drawn fibre

For the longest fibre available, the rotational ϕ angles at which the {220} and {422} Bragg reflections (the only two occurring) were observed were recorded and these are plotted in Fig. 4a. For the first 60 mm, the scan window was reduced to 5 mm, while for the separate piece from a position 150 mm away, the slit width was 15 mm. Error bars are indicative of the uncertainty of the $\phi = 0$ placement. The relative $\phi = 0$ position for the final piece was not known, but no rotation was seen among the other pieces measured. The box plot (Fig. 4b) presents the angular difference between the phi values for the two peaks, along with the expected observation for their separation based on a <111> alignment along the axis.

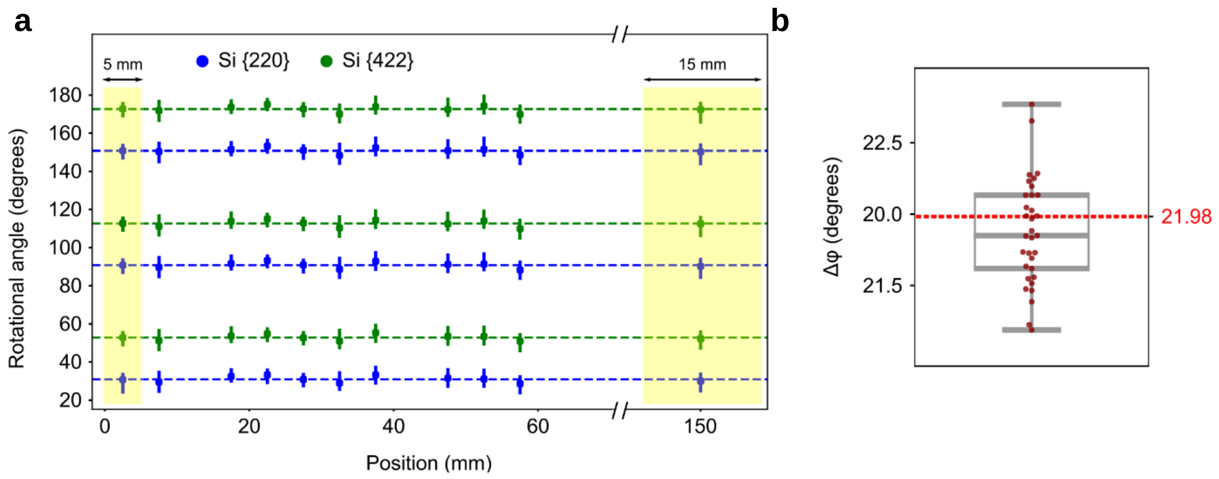

Fig. 4 Rotational angles at which Bragg peaks are observed (for the first 180 degrees), for several pieces of fibre taken from a continuous 190mm length. a) Green curves are for the {422} reflections, and blue are for the {220}, showing the angular positions and separations of the peaks are constant. Dashed lines are average values. b) box plot of the angular separations for the two reflections, showing values tightly clustered around the theoretical value of 21.98 degrees (shown in red)

Supplementary Note 4: EBSD of as-drawn GaSb/Si core

Fig. 5 illustrates the epitaxial relationship between the Si and GaSb in the as-drawn fibre. Fig. 5a is an EBSD image of a side polished fibre and Fig. 5b is an SEM of the same area, showing clearly the pseudo-eutectic inclusions. The orientation colour maps, shown in Fig. 5c are identical for the two lattices, and EBSD analysis reveals a single crystallographic orientation across the boundaries between the two materials, with only minor inclusions of different orientation.

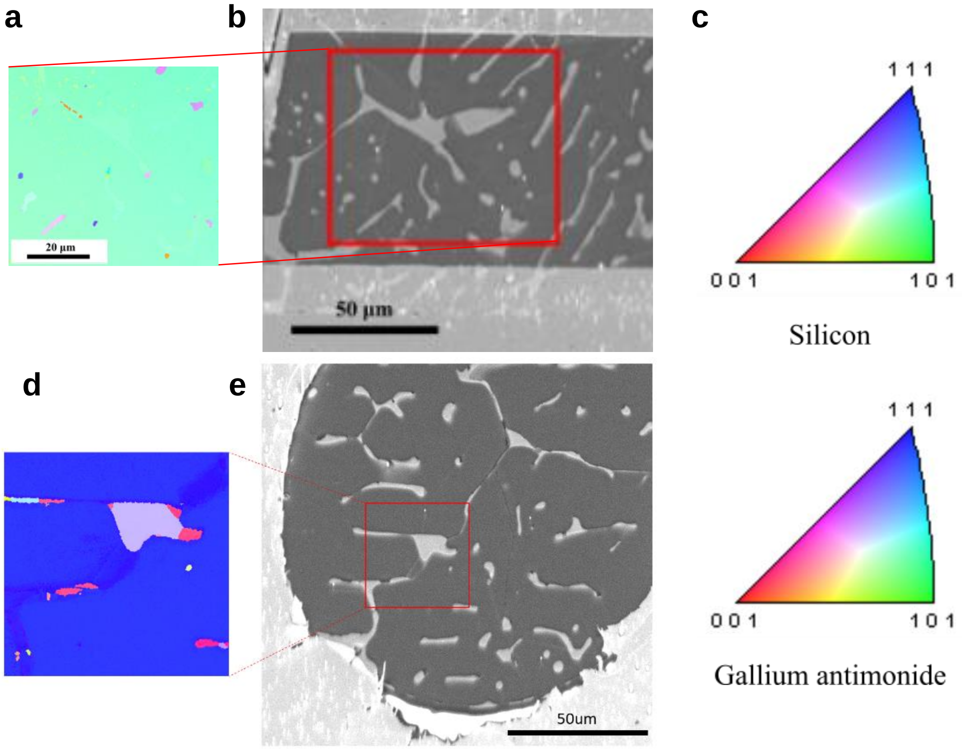

Fig. 5 EBSD of as drawn fiber (a) orientation map of side polished fiber, (b) SEM image of the fiber (c) orientation maps for Si and GaSb, (d) orientation map for cross-section of a fiber, showing misaligned GaSb inclusion, and (e) SEM of entire cross section

Fig. 5d is an EBSD map of a cross-section of one of the as-drawn fibres, and the associated SEM image is shown in Fig. 5e. The fiber cross-section has the {111} orientation anticipated by XRD results. There is one large misaligned grain in this sample, indicating that some of the XRD texture observed in the GaSb arises from structures in the interior of the core.

Supplementary note 5: EBSD of segregated fibre

Fig. 6 contains images from the segregated regions of the fibre. Fig. 6a is a secondary electron image of the pure silicon region, and Fig.6b shows that this silicon, left after the removal of GaSb, is all one orientation. The specks of a different colour are contaminant particles, visible in the SEM image.

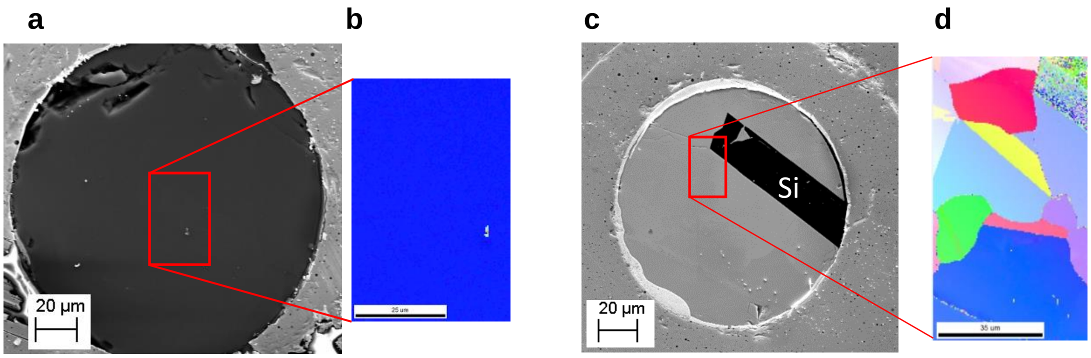

Fig. 6 SEM and EBSD images of silicon and GaSb cross-sections of a segregated fiber. (a) SEM of silicon region (b) EBSD map of the silicon, showing the (111) orientation calculated using the XRD data. (c) Cross section at the transition to the GaSb area, showing incomplete Si segregation

Fig. 6c is an SEM image of GaSb in the transition region between the two compositions, showing a Si crystal that penetrates into the GaSb. As seen in the EBSD of Fig. 6d, the GaSb is polycrystalline, confirming the XRD results shown below. The solidification of the GaSb region occurs rapidly, in part due to the digital laser control, and in part because there is a single temperature at which the transition occurs. The rapid cooldown leads to formation of multiple seeds and a polycrystalline structure. The upper right-hand corner of the EBSD image is noisy, due low contrast in the Kikuchli lines, hindering clear orientation determination.

Supplementary note 6: {111} peak X-ray results for segregated fibre

Fig. 7 shows the rotational scan data from the GaSb in a polycrystalline GaSb fibre, an annealed composite fibre, where the GaSb {111} peaks appear at many angles, indicating polycrystalline solidification, and the {111} signal before laser scanning, where the {111} orientation is barely measurable. It is important to note that this signal is derived from the outside ~20 μm of the sample due to the large absorption of Mo X-rays by GaSb, and Figure 2(f) in the main text shows that there is still epitaxial alignment of the Si and GaSb.

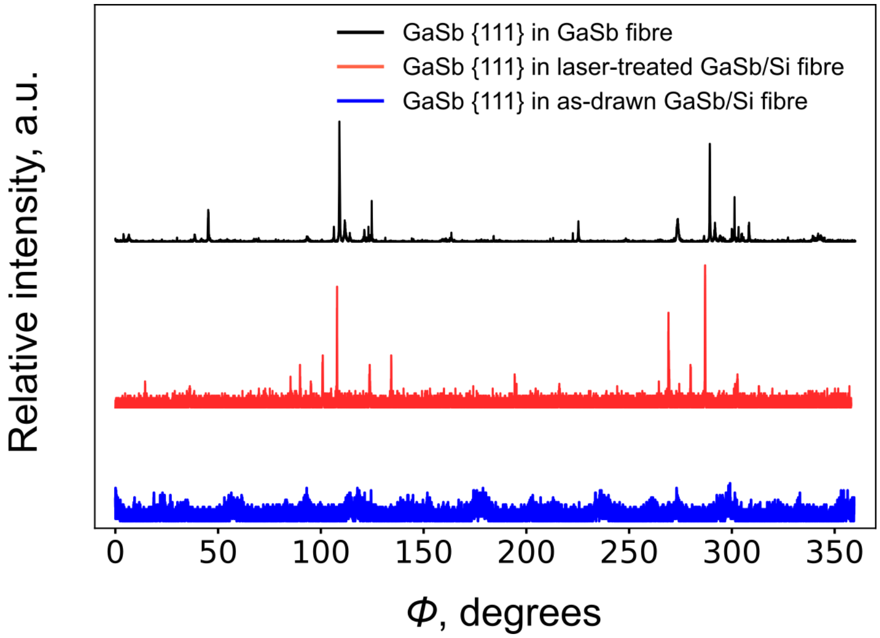

Fig. 7 XRD results for GaSb {111} peaks in pure as-drawn GaSb fiber, GaSb in the aggregated region of a laser treated fiber, and GaSb in the as-drawn fiber.

Supplementary Note 7: X-ray analysis using capillary Bragg instrument

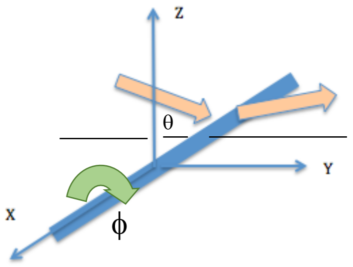

Fig. 8. Geometry for X-ray diffraction

Normally, diffraction equipment with a capillary sample holder is used to identify small quantities of unknown minerals in powder form. Rotation of the capillary around its longitudinal axis assures the capture of all possible reflections for each Bragg angle, as shown in Fig. 8.

In contrast, for the fibre cores studied here, there can be three-dimensional crystalline order over large regions, in which case a particular Bragg peak appears only for a small number of rotational positions *(ϕ)* of the fibre. Measurement of the angles between those rotational positions allows determination of symmetry and orientation for a single crystal, and estimation of the number of crystallites in the excitation volume if the sample is polycrystalline.

For a single crystal, with θ-2θ chosen for a particular d-spacing, rotation in ϕ will result in reflections at separations that are determined by the crystal symmetry. As an example, in the cubic system, the {220} reflections appear at six values of ϕ, separated by 60° if a <111> axis coincides with that of the fibre, as shown for a pure silicon fibre in Fig. 9.

Generally, several families of planes yield reflections, for a single crystal. The angular separation of the Bragg reflections allows determination of the axial orientation. Combining the ϕ responses for several θ-2θ peaks on one set of axes provides an overview of the structure. For the analysis of these fibres, the

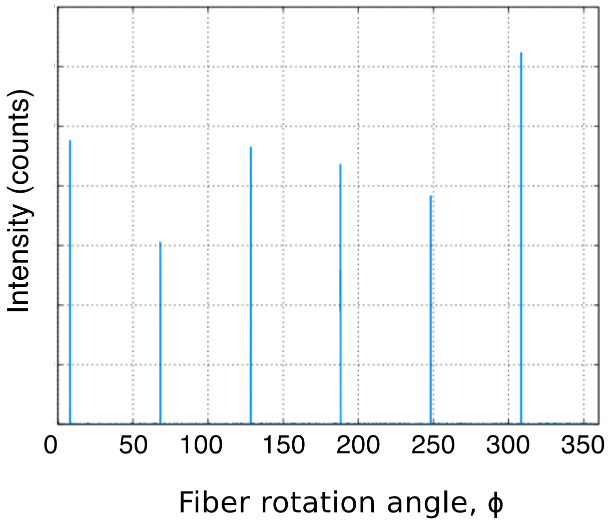

Fig. 9 Rotational (ϕ) scan for the {220} reflection of an annealed intrinsic silicon fibre, showing six-fold symmetry associated with <111> alignment along the fibre axis. Intensity variations are indicative of imperfect alignment with the instrument measurement axis. The narrow FWHM of the peaks indicates excellent crystalline order over the ~1.5 cm covered by the beam.

possible values for the ϕ separation of the different Bragg peaks were determined from the dot products of the Miller indices of the observed reflections. To find all possible ϕ separations within a family of planes ($\Delta\phi_1$), both sets of indices are permuted considering both positive and negative values. For analysis of interfamily angular separations ($\Delta\phi_2$), corrections must be made for offsets resulting from the movement of the source/detector for different Bragg reflections. In the case where a unique (including inversion) combination of indices satisfied all of the angular separations observed, a cross product of the indices for the observed planes yielded the crystallographic orientation of the fibre axis. This general method can be applied to more complex situations, as shown below.

For some of the early-drawn GaSb/Si fibre, axial directions of <211> and <433> were observed. Fig. 10 shows a fibre with the <433> direction aligned with the fibre axis. $\Delta\phi_1$ is the angle difference between the two {3 3 1} planes, and it was measured to be 37.69°. This angle matches the calculated angle between the (331) and (313) planes of a cubic crystal. In order to calculate an angle between {2 2 0} and {3 3 1}, the angle at which the data is acquired must be considered in addition to the measured $\Delta\phi_2$, as the effective zero for ϕ is shifted by θ when the detector moves to acquire a new Bragg peak. For the example here, the change in recorded ϕ is 5.89° because the {2 2 0} peak appears at θ=10.65° and the {3 3 1} peak appears at θ=16.54°. $\Delta\phi_2$ was measured to be 64.78°; with the correction, this gives 70.67°, which coincides with one of the calculated dot products.

The ϕ separations from the scan of the GaSb/Si fibre match $(0\ 2\ \bar{2})$ and (3 3 1), planes in a single crystal. The axial orientation of the core is then $[\bar{4}\ 3\ 3]$ from a vector product of $[0\ 2\ \bar{2}]$ and [3 3 1]. The relative intensities of the peaks and the phi scans were the same for three samples taken from a 100 mm fibre, indicating that the crystal orientation, once established, is stable for some time.

While the angular separation of peaks is reliable for analysing these fibres, peak heights are not, as misalignments of the fibre axis of a few degrees can alter the observed amplitudes significantly.[1]

For the highly oriented fibres discussed in this paper, there were only two independent families of planes observed, the {220} and the {422}, simplifying the analysis. The six-fold symmetry for both families confirmed the calculated <111> axial alignment.

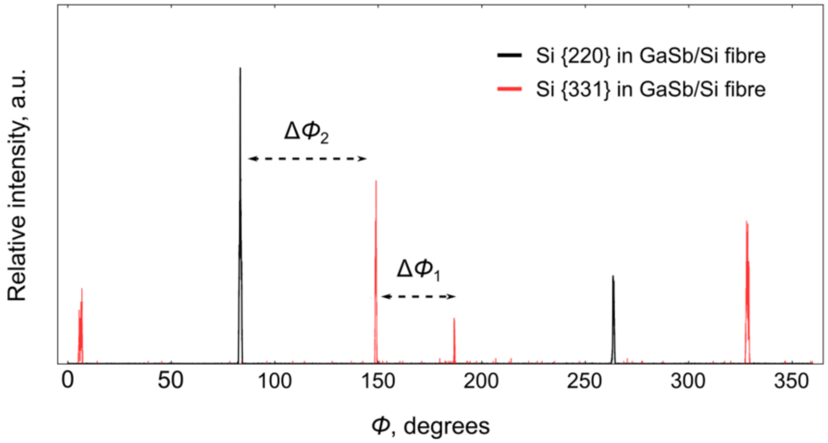

Fig. 10 Axial rotational X-ray scan with the detector set at 2θ for the {220} and {331} planes for silicon in an early GaSb/Si-core fibre. The values of $\Delta\phi_1$ and $\Delta\phi_2$ were used to determine the crystalline orientation of the core material.

Supplementary References

1. Kristin Lønsethagen. Recrystallization of Si-core glass fibers by laser annealing. (NTNU MS thesis, 2017).
2. Nordstrand, E. F., Dibbs, A. N., Eraker, A. J. & Gibson, U. J. Alkaline oxide interface modifiers for silicon fiber production. *Opt. Mater. Express* **3**, 651–657 (2013).
3. Yan, Y. L., Wang, Y. X. & Zhang, G. B. A key factor improving the thermoelectric properties of Zintl compounds A(5)M(2)Pn(6) (A = Ca, Sr, Ba; M = Ga, Al, In; Pn = As, Sb). *Comput. Mater. Sci.* **85**, 88–93 (2014).
4. Johnson, S. I., Zevalkink, A. & Snyder, G. J. Improved thermoelectric properties in Zn-doped Ca5Ga2Sb6. *J. Mater. Chem. A* **1**, 4244 (2013).
5. Young, D. & Kauzlarich, S. Preparation, Structure, and Electronic-Properties of Ca(11)msb(9) (m=al, Ga, In). *Chem. Mater.* **7**, 206–209 (1995).
