# Supplementary material for: Laser restructuring and photoluminescence of glass-clad GaSb/Si-core optical fibres
Source: Nat Commun. 2019 Apr 17;10:1790. doi: 10.1038/s41467-019-09835-1 (PMC6470204; doi:10.1038/s41467-019-09835-1)
Supplement: Supplementary file 3 — Description of Additional Supplementary Files [file 41467_2019_9835_MOESM3_ESM.docx]

**Title:** Supplementary Video 1

**Description:** This video shows the initial stages of the aggregation of GaSb when the fibre is illuminated with a CO_2_ laser (incident from the top of the image). The final three seconds of the image show the translation (fiber moving right to left).
